# The relationship between digital transformation and digital literacy - an explanatory model: Systematic literature review

**DOI:** 10.12688/f1000research.146991.1

**Published:** 2024-04-05

**Authors:** José Arnaud, Henrique São Mamede, Frederico Branco

**Affiliations:** 1 Instituto Politecnico do Cavado e do Ave, Barcelos, Braga, 4750-810, Portugal; 2 Institute for Systems and Computer Engineering Technology and Science, Porto, Porto District, 4200-465, Portugal; 3 Universidade Aberta, Lisbon, Lisbon, 1269-001, Portugal; 4 Universidade de Tras-os-Montes e Alto Douro, Vila Real, Vila Real, 5000-801, Portugal

**Keywords:** Digital Literacy; Digital Transformation; Explanatory Models; Systematic Literature Review

## Abstract

Digital transformation has been one of the main trends in organizations in recent years, and digital literacy is a critical factor in the success of this transformation. Digital transformation involves the use of digital technologies to improve an organization’s processes, products, and services. For this transformation to be successful, it is necessary for employees to have knowledge of and skills in digital technologies. Digital literacy allows employees to understand technologies and their applications, know how to use them efficiently and safely, evaluate and select the most appropriate digital tools for each task, and be prepared to deal with problems and challenges that arise in the digital environment. Thus, this study is relevant because it seeks to understand how digital literacy can impact Digital Transformation in organizations and, through the construction of an explanatory model, allows the identification of variables that influence this relationship by developing strategies to improve the digital literacy of employees in organizations.

## 1. Introduction

According to Datta (2020) and Blyznyuk et al. (2021), Digital Transformation (DT) positively impacts people’s daily lives and simplifies organizations, but for this to happen, people and organizations must be willing to change and restructure their attitudes towards DT and then the way they work. According to Ivanova and Putintseva (2020), DT allows for a change in society in terms of relationships with organizations. Currently, DT is a necessity for any organization, whether public or private, because of the vertiginous acceleration that digitization has had in society, which has meant that many organizations have not yet adapted to DT ( Alvarenga et al., 2020). Although most organizations are already going through the DT process by incorporating digital technologies, many challenges must be overcome before fully embracing DT ( Rupeika-Apoga et al., 2022; Zhou et al., 2023). According to Tanushev (2022), successful organizations in DT are more productive, efficient, technologically advanced, and therefore more competitive, digital technologies are quite broad ( Schneider & Kokshagina, 2021).

The COVID-19 pandemic saw an acceleration of DT in organizations, helping lay the foundation for the implementation of this transformation ( Gabryelczyk, 2020). The same author stated that the pandemic forced an acceleration in the expansion of Information Technology (IT) infrastructure, as it became clear that resources were scarce. As for Al-Alawi et al. (2023), COVID-19 made remote work the new normal, which meant that organizations had to ensure that their employees had the technology to carry out their professional activities. However, resistance to change is an important challenge in DT. COVID-19 has created a range of opportunities for innovation and transformation in organizations, associated with the use of Information and Communication Technologies (ICT) ( Alam et al., 2022).

DT adopts technology to transform services or business, replacing manual processes with digital processes, or upgrading existing technology with newer technology ( Boban & Klaric, 2021; Park, Lee & Chung, 2022; Stoumpos et al., 2023). Most modern information technologies offer new opportunities for greater efficiency in the quality of service of organizations; however, the resulting benefits may not be guaranteed depending on several factors, such as accessibility, adaptability, and specialized personnel ( Surnin et al., 2021).

During DT, the main organizational processes are redesigned, new technological tools replace older tools, new capabilities are developed, and new ways of working are implemented ( Pittaway & Montazemi, 2020; Scupola & Mergel, 2022). Organizational size is the first variable to consider when discussing organizational change ( Tangi et al., 2020). DT requires organizations to adopt new strategies, organizational transformation, and new ways of implementing strategies ( Rêgo et al., 2022), which are mainly driven by organizational flexibility ( Sergei et al., 2023) thus improving their competitiveness ( Yu et al., 2022) and increasing their productivity and efficiency ( Balashov et al., 2020).

According to Hanelt et al. (2021), DT can be defined as the organizational change triggered and shaped by the widespread diffusion of digital technologies. Thus, DT can be defined as “the most profound and accelerating transformation for business activities, processes, competencies, and models to leverage the changes of digital technology and their impact in a strategic and prioritized way” ( Hamidi et al., 2018, p. 723).

The definition of DT can be broad, ranging from the profound transformation of organizational activities to the advanced use of digital technologies.

DT will be a process of changing mindsets towards the use of technology, as people are at the center of DT and are responsible for its success. It is important, then, to make people part of the process, assuming them as a key factor of change; otherwise, people will not be part of the solution but part of the problem.

People, processes, and technology are the three most important vectors in DT; however, if the digital literacy (DL) of the people involved is scarce, this could jeopardize this transformation. According to Fernández et al. (2023), people are the most essential part of an organization; therefore, it is very important to ensure their involvement and training when implementing DT. According to Nadkarni and Prügl (2020), all people who are part of an organization, regardless of their role or position, play a key role in the DT process, whether due to the simple need to acquire technical skills or the desirable involvement in taking advantage of all the potential of the adopted technologies, so that they can participate in the inevitable organizational transformation in these processes. The authors also refer to the problem of a possible division between older collaborators and less integration in the changes, in contrast with the new ones, which are more adapted to the technologies being adopted. The need to empower older people and adapt models and their usability are critical elements that need to be addressed for a better DT ( Zanutto, 2021; Lee et al., 2022). According to Bykov and Medvedera (2021), DL among the younger population is generally present.

Currently, people are the most important resources in organizations because without them, organizations do not exist; however, to have quality and motivated employees, it is necessary to train them with the digital skills necessary to perform their work effectively ( Glavas, Uroda & Mandic, 2021; Grgurevic et al., 2022; Galushi & Malatji, 2022; Jonathan et al., 2021). This need for digital skills was quite evident during the COVID-19 pandemic, as many people felt the need to find a new job or keep their current job, as organizations needed employees qualified in digital skills ( Stofkova et al., 2022). The authors also add that some digital knowledge was optional a few years ago, was not considered important, and soon became critical.

It is necessary to reinforce the sharing of digital skills among employees during the DT process ( Dee et al., 2019; Bousdekis & Kardaras, 2020). According to Shin and Rakhmatullayev (2019), Francisco et al. (2019), and Datta et al. (2020), the need for training is very important for those who do not have DL.

The most popular DL concept is that of Paul Gilster. Gilster (1997). He states that DL requires a profound change in the way we face the concept of literacy, and that it forces us to rethink the way we locate, evaluate, organize, read, and write information. Gilster defines DL as “the ability to understand and use information in multiple formats from a wide variety of sources when it is present via computers.” ( Gilster, 1997, p.1). Tabusum et al. (2014) defined DL as the ability to locate, organize, understand, evaluate, and analyze information using technology. The same author states that having DL is not just knowing how to use the computer, but knowing how to use digital technologies to communicate. Walton (2016) defined DL as the ability to find, evaluate, use, share, and create content using the Internet and ICT. Based on these authors, we can say that DL is a vital competence in the knowledge society, but that, at the same time, it can constitute a barrier to personal development and social integration if it is absent or underdeveloped in the population.

The definition of DL changes according to different authors because new technologies and technological innovations change how people use technology and perform tasks. DL implies that the user can use information constructively.

This theme brings important reflections about DL because with technology and processes being transformed, the discussion on DL is very important.

According to Feher and Szabo (2019), to digitally transform companies, they must transform their internal processes and their processes with their customers and business partners. The same authors also mentioned that company employees must be able to use technology and be digitally literate, as these skills are not just for the IT team. They add that organizations cannot transform overnight, and that the organizational culture itself and its employees cannot accept this type of DT.

Al-Alawi et al. (2023) implemented well-established employee training and coaching as DT projects are subject to failure if employees adopt a passive position. This type of position on the part of employees can have consequences contrary to what was expected, that is, the relief of work that should exist with DT does not happen. Instead, we experience increased stress, increased workload, more time to process each case, and more demanding procedures ( Kuhlmann & Heuberger, 2023). To create sustainable and meaningful DT, employees of organizations will have to play a critical role in the adoption and application of new technologies in different areas of the business ( Ahn & Chen, 2022). According to Antunes (2022), it is essential to understand the relationship between knowledge and DT. DT has become a constant, opening countless possibilities to enrich the digital knowledge of employees in organizations, forcing them to continually update their digital skills, which is why DL is a prominent topic ( Farias-Gaytan et al., 2022).

Digitally transforming an organization may imply that people must be prepared and equipped with tools and, in this way, be the main drivers of this transformation. This makes it possible to rethink the processes associated with this transformation and use the added value of technology to carry out the respective implementations. Therefore, it is extremely important to reflect on the current state of digital skills in our society, more specifically, the digital skills of employees in organizations, and propose reforms and structural measures that are the true basis of sustainability.

An organization’s DT involves the implementation of digital technologies in all processes and business areas, which can lead to a significant change in the way activities are carried out and how teams collaborate. However, for DT to be successful, it is essential that the organization’s employees have an adequate level of DL, that is, skills and competencies to effectively use digital technologies in their work. Therefore, the development of an explanatory model that relates DT to the DL of employees is crucial for the organization to be able to plan and implement strategies for developing digital capabilities, ensuring that all employees can make the most of the technologies. digital implemented. This can lead to greater DT success and an overall improvement in the organization’s efficiency and productivity.

Kleinman et al. (1978) introduced the concept of the explanatory model and referred to the complex and culturally influenced process of interpretation that people make in relation to their own illness. This process includes giving meaning to symptoms, assigning causes to illnesses, and formulating expectations about treatment and outcomes. In summary, the explanatory model is a way in which everyone understands and explains their condition and health. Thus, explanatory models are simplified representations of reality that allow one to understand and explain the relationships between different variables that affect a phenomenon. Explanatory models are used to predict the behavior and outcome of a phenomenon and identify the variables that are most important for its occurrence. Several authors have addressed the concept of explanatory models in different areas of the study. According to Aznar-Díaz et al. (2020) and Romero-Rodríguez et al. (2022), explanatory models can provide information on aspects that can influence the development of good pedagogical practices.

In the area of technological innovation, there are also authors who address the concept of explanatory models, such as Ferreira and Torres (2017), who address the process of technological innovation in organizations and present different explanatory models to understand this process. The authors discuss important concepts, such as innovation, technology, explanatory models, and the relationship between them.

Thus, the importance of research and development of an explanatory model to understand the relationship between DT and DL of an organization’s employees is highlighted. It is critical that organizations understand how the DL of their employees can impact the organization’s DT, as this understanding can lead to more effective implementation and continuous improvement of DT. SLR is an important approach to identify and analyze relevant studies on DT and DL, and the description of these concepts can provide a solid basis for the development of an explanatory model. The scarcity of research investigating the relationship between these themes makes the need to conduct this research even more relevant.

By exploring the impact that DL can have on DT, an explanatory model can help organizations develop more effective strategies to improve the DL of their employees, thereby increasing the success of DT. This can lead to an increase in the efficiency, productivity, and competitiveness of organizations.

The remainder of this paper is organized as follows. In the next chapter, we present the methodology used for the Systematic Literature Review process. We then analyze the results obtained to answer the research questions and present some results obtained in relation to each of these questions, providing a more comprehensive understanding of the study. Finally, we discuss the theoretical and practical contributions before concluding the main findings and possible future research.

## 2. Method

**Figure 1.**
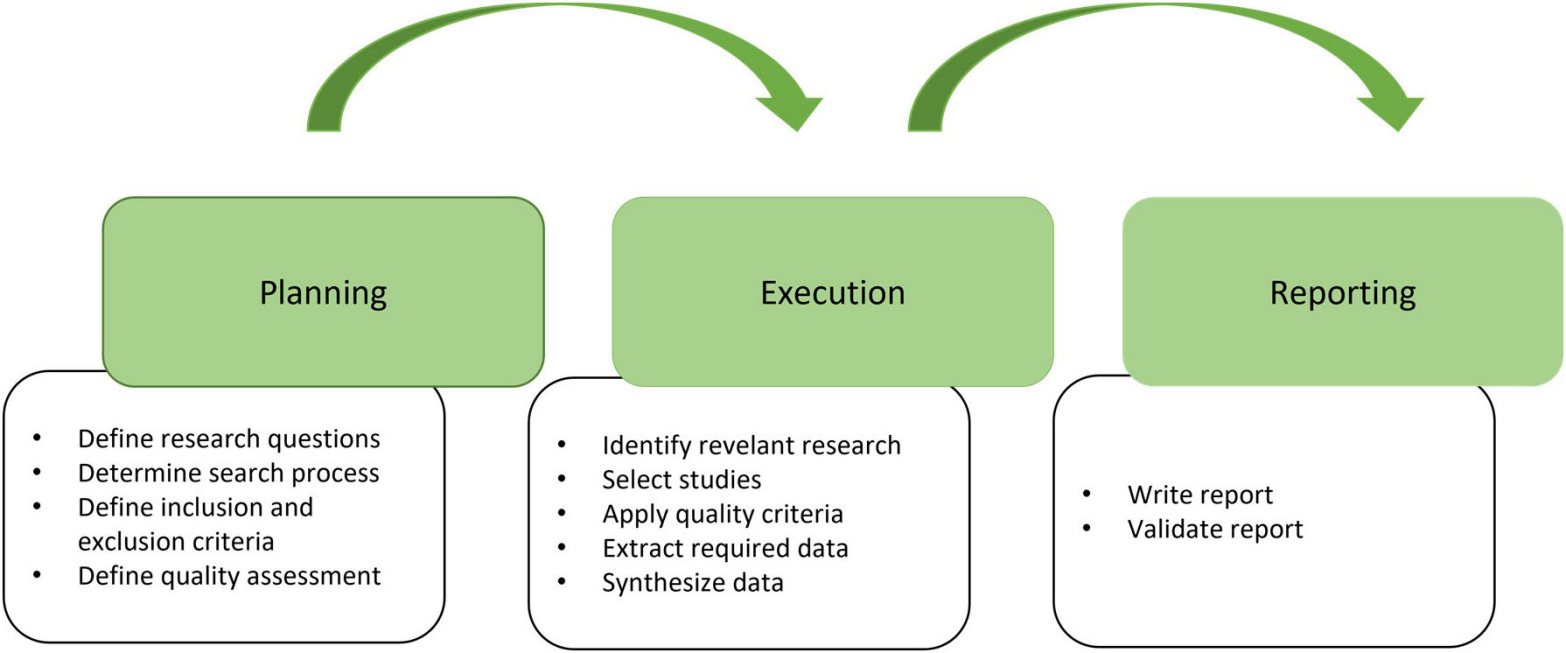
Systematic Literature Review process activities (adapted from Kitchenham (2004)).

In this study, we followed Kitchenham’s guidelines (2004), involving several activities, as shown in Figure 1 and is grouped into three main phases: Planning, Execution and Reporting.

• **Planning phase**: In planning phase, the objective to be achieved by the Systematic Literature Review (SLR) is defined, which includes formulating the research question and defining the inclusion and exclusion criteria for the studies to be selected. At this stage, it is also important to define the search terms and databases used in research. Finally, the quality of the retrieved papers was assessed.
• **Execution phase**: In execution phase, a systematic search for relevant studies is carried out according to the inclusion and exclusion criteria defined in the planning phase. Documenting the study selection process to ensure review transparency and replicability is important. In this phase, the selected studies were evaluated for their quality and relevance to the research questions.
• **Reporting phase**: In reporting phase, data are synthesized and analyzed to answer the research questions. This phase included extracting relevant data from selected studies, analyzing the data, and interpreting the results.

## 3. Results

The research questions and their formulations must be chosen according to the focus and objective of the SLR. In this case, the research objective seeks to understand how DT impacts the DL of employees in local public administration, identifying the main factors that influence this relationship. This may include an analysis of the digital skills needed, the training and capacity building, and the barriers faced by employees in the use of digital technologies, among other relevant aspects. By proposing an explanatory model, this study aims to provide a solid theoretical framework that helps understand how DT affects the DL of employees in local public administration, thus enabling the development of more effective strategies to promote digital empowerment and the successful adoption of digital technologies in this specific context.

To assist in the construction of the research questions and their search for evidence, it was necessary to define five fundamental points, namely, Population, Intervention, Comparison, Outcome, and Context (PICOC), as shown in Table 1.

**Table 1.** PICOC (Population, Intervention, Comparison, Outcome, Context).

**Population**	Local public administration employees
**Intervention**	Digital transformation
**Comparison**	There may not be a direct comparison, as the focus is to explore the relationship between DT and DL of the employees
**Outcome**	DL of local public administration employees
**Context**	Local public administration

To begin the research, a strategy for searching articles was defined. Thus, in addition to the existence of other digital libraries used in an SLR, depending on the topic’s interdisciplinarity, the following are suggested:

• Scopus – http://www.scopus.com
• Science Direct – http://www.sciencedirect.com
• IEEE – http://ieeexplore.ieee.org
• Springer – http://link.springer.com

We structured the research questions using logically organized keywords. To this end, it was necessary to define search strings with the terms to be searched in a way that contemplates the research questions. The logical functions “AND” and “OR” were used to concatenate words, as digital libraries support this type of chaining. It is important to emphasize that the process of defining the search strings is iterative and involves several cycles of experimentation, checking the articles found, and adjusting the search strings. String adjustment is relevant to gain more robustness and retrieve the most relevant primary articles ( Kitchenham and Charters, 2007).

The search strings used were as follows:

• TITLE-ABS-KEY ((“explanatory models”) AND (“techniques” OR “methods” OR “approaches”)).
• TITLE-ABS-KEY ((“digital transformation”) AND (“review” OR “survey”))
• TITLE-ABS-KEY ((“digital literacy”) AND (“review” OR “survey”))
• TITLE-ABS-KEY ((“digital transformation” AND “digital literacy”))

The main concepts of this research were Digital Transformation, Digital Literacy and Explanatory Models. Four digital libraries were used in view of the scope of the search terms’ results, and a significant number of articles were found through the search strings. Table 2 shows the research analysis model carried out on the main concepts during the months of March and April 2023.

**Table 2.** Research in digital libraries.

ITEMS	SCOPUS	SCIENCE DIRECT	IEEE	SPRINGER
**SEARCH STRINGS**	TITLE-ABS-KEY ((“explanatory models”) AND (“techniques” OR “methods” OR “approaches”)) TITLE-ABS-KEY ((“digital transformation”) AND (“review” OR “survey”)) TITLE-ABS-KEY ((“digital literacy”) AND (“review” OR “survey”)) TITLE-ABS-KEY ((“digital transformation” AND “digital literacy”))			
**ARTICLES FOUND**	7.492	21.492	894	27.621

After the search strings were structured and validated, it was necessary to delimit the criteria used to exclude and include the articles that were found. The purpose of defining the criteria is to identify the primary studies that provide direct evidence for the research questions ( Kitchenham & Charters, 2007).

Exclusion and inclusion criteria were applied to each article found in searches of digital library repositories, with some of the criteria defined based on practical issues of publications, such as the language in which it is written, type of publication, and year of publication. others. Secondary articles, summarized articles, technical reports, non-peer-reviewed publications, and redundant articles were excluded. If in the exclusion criteria it was enough for only the articles to be included in one of the criteria to be excluded from the review, in the inclusion criteria all the criteria had to be satisfied to be included in the final list of articles. For example, the timeframe entered in the search was delimited as an inclusion criterion. Another example of an inclusion criterion is the criterion of being only primary and peer-reviewed articles. The inclusion criteria are listed in Table 3.

**Table 3.** Inclusion and exclusion criteria.

ITEMS	SCOPUS	SCIENCE DIRECT	IEEE	SPRINGER
**INCLUSION CRITERIA**	Studies from 2018. Primary studies. Studies on explanatory models. Studies on DT. Studies on DL. Peer-reviewed studies.			
**EXCLUSION CRITERIA**	(1) Studies prior to 2018. (2) Duplicate studies. (3) Studies in any language other than English. (4) Studies in any language other than Portuguese. (5) Secondary or tertiary studies. (6) Literature such as manuals, reports, theses, dissertations. (7) Short papers (4 pages or less).			
**EXCLUDED ARTICLES (1)**	2.171	9.182	268	10.817
**EXCLUDED ARTICLES (2)**	34	546	11	689
**EXCLUDED ARTICLES (3, 4)**	526	365	84	250
**EXCLUDED ARTICLES (5)**	4.616	10.720	382	14.818
**EXCLUDED ARTICLES (6)**	24	78	14	96
**EXCLUDED ARTICLES (7)**	75	599	129	951
**SELECTION FILTERS**	Title and Abstract and/or Introduction and Conclusion and/or Full Reading			
**ARTICLES ANALYSED**	46	2	6	0

To obtain the final articles to be analyzed, the exclusion criteria were applied to 57.499 articles obtained initially. Table 4 shows the number of articles analyzed using selection filters. The number of articles in which the title and abstract were used, the number of articles in which the introduction and conclusion were used, and the number of articles in which there was full reading.

**Table 4.** Selection Filters.

SELECTION FILTERS	SCOPUS	SCIENCE DIRECT	IEEE	SPRINGER
**Title and Abstract**	4	0	1	0
**Introduction and Conclusion**	10	0	2	0
**Full Reading**	32	2	3	0

**Figure 2.**
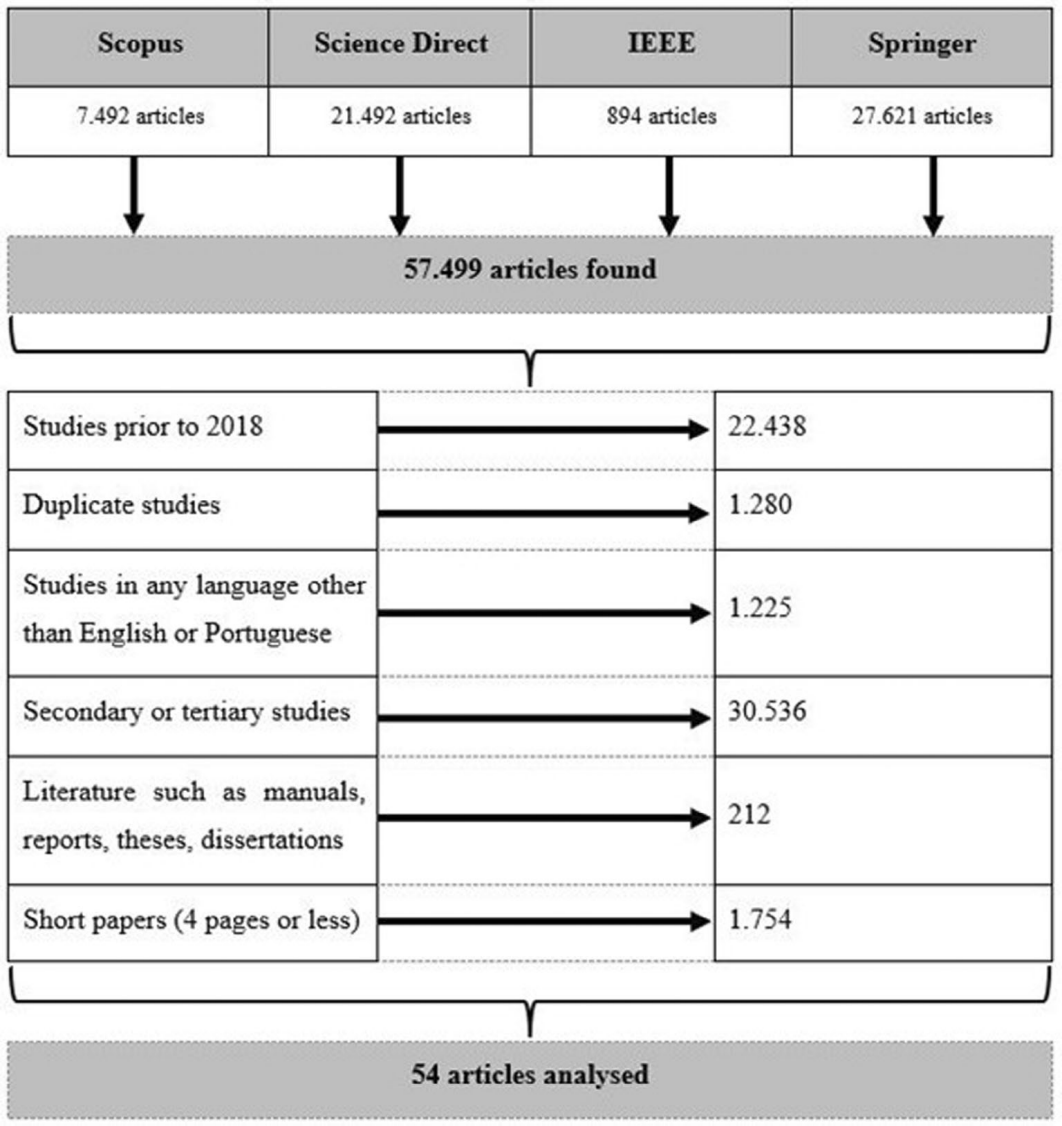
Papers filtering (excluded articles).

Thus, Figure 2 summarizes the excluded articles in the four digital libraries.

**Figure 3.**
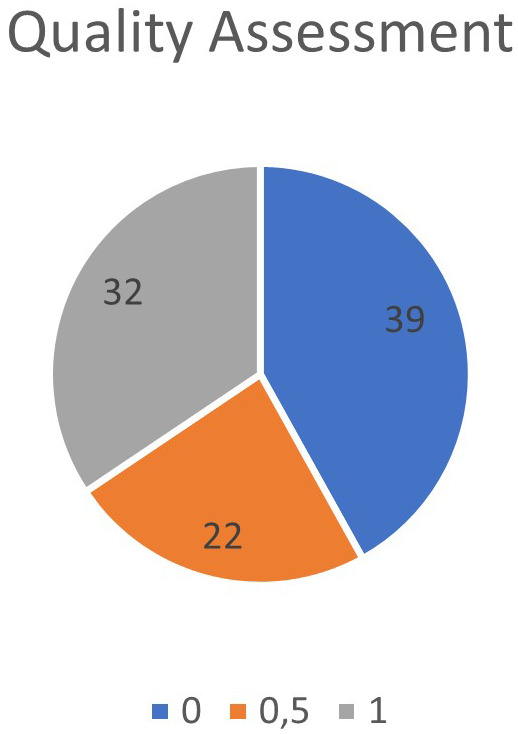
Quality of articles selected for data extraction.

**Figure 4.**
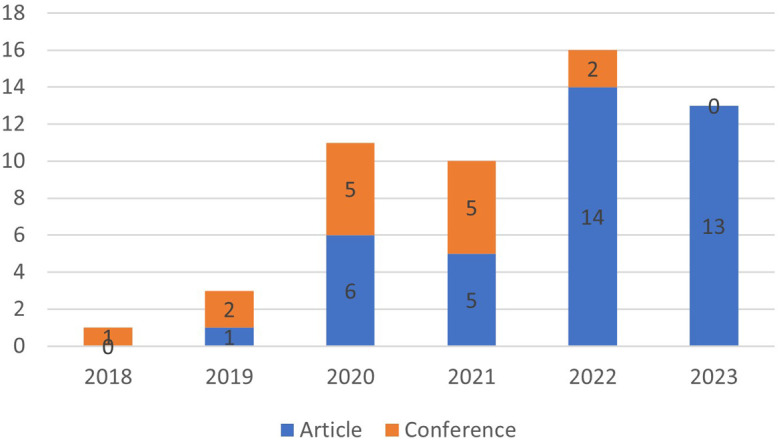
Year of publication of articles selected for SLR.

After applying the exclusion criteria and defined criteria to assess the quality of the articles, it was found that 39 articles did not contain any criteria as defined, 22 articles contained some criteria as defined, and 32 articles met the defined criteria, as shown in Figure 3. In Figure 4, the year of publication of the articles selected for SLR was verified.

After performing a quality assessment and obtaining a set of 54 articles valid for the SLR, it was necessary to collect data from these articles to answer the research questions.

To date, we have been able to identify 54 articles in the traditional review model and 93 articles in the snowballing model. The most cited article had all its references in English, and in one specific case, an article appeared as a result of three different strings.

These data, being an SLR, must be carefully extracted. It is important to extract and organize the most general data from the articles, such as the title of the article, authors of the article, year of publication of the article, country of the article, and type of publication of the article (for example, if it was published in a conference or if it is a chapter of a book), among others.

Thus, 54 articles have the essential criteria to be analysed for data extraction.

After completing the search and selection of articles, a set of articles was selected, which served as the input for the next steps of the SLR protocol. One of the steps is the quality assessment of these articles, which is useful for increasing the accuracy of the data extraction results, thus enabling greater credibility of the results.

Kitchenham and Charters (2007) argued that the execution of the quality assessment of the included studies is critical for the following reasons:

• Provide even more detail to apply exclusion/inclusion criteria.
• Investigate whether quality differences explain differences in article results.
• Quality assessment is a way to give more value to the importance of individual articles when the results are synthesized.
• This step can guide the interpretation of results and determine the strength of inferences.
• Can guide the recommendation of future work.

In an SLR, several criteria can be used to measure the quality of articles. Kitchenham and Charters (2007) referred to several sources that can help define the quality criteria that can be evaluated. These criteria are usually based on instruments containing checklists of factors, normally using a numerical scale that must be evaluated in each article. In this case, the scales of 0, 0.5, and 1. Scale 0 indicates that no criterion is in accordance with what was defined; the 0.5 scales to define that some criteria are in accordance with what was defined; and scale 1 indicates that the criteria are in accordance with what was defined.

Several issues can be considered when assessing the quality of research articles. It is important to note that the relevance and weight assigned to each of these issues may vary depending on the context of the research and the specific criteria of the review. Some of the questions and their relevance are listed in Table 5.

**Table 5.** Questions and their relevance.

QUESTIONS	RELEVANCE	VALUE
Author qualification	Are the authors' experience and credits relevant to the field of study?	0.5
Reliable sources	Have the articles been published in reputable peer-reviewed scientific journals?	1
Methodology	Is the methodology used clearly described and appropriate for the research question?	1
Sample	Is the sample representative and sufficiently large? Are the criteria for selecting participants appropriate?	1
Validity and reliability	Does the study use valid and reliable measures and instruments? Are strategies to ensure internal and external validity mentioned?	1
Data analysis	Is the quantitative or qualitative analysis appropriate and rigorous? Are the results presented clearly and are they consistent with the data collected?	1
References	Are the articles cited relevant, current and do they cover the relevant literature?	0.5
Contribution to the study	Does the study offer new perspectives, insights or significant contributions to the field of study? Do the results have relevant practical or theoretical implications?	1

Regarding the exclusion of articles, during the review process, exclusion criteria can be established based on the specific aims and criteria of the research, with the value to be assigned being zero (0). Some common criteria may include direct relevance to the research questions, methodological quality, lack of relevant data or lack of significant contribution to the field of study. It is important to note that the assessment of the quality of articles is a subjective process, and that the final selection of articles should be made based on a combination of criteria and critical judgment.

**Figure 5.**
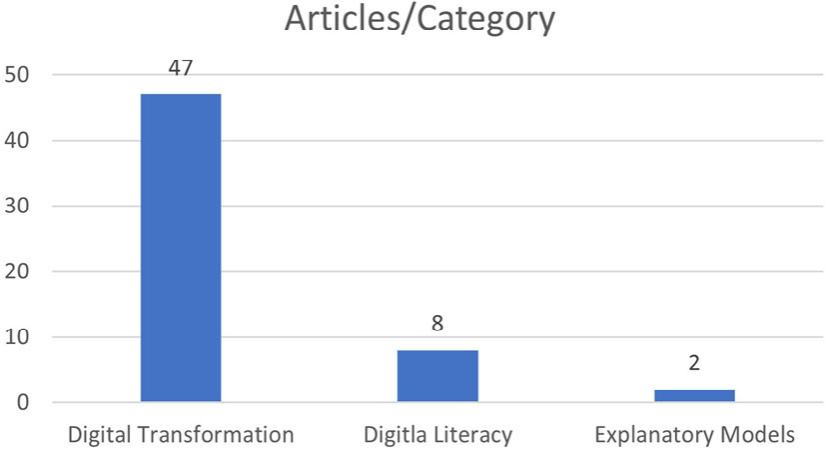
Number of articles selected by category.

A total of 54 articles were selected for analysis and data extraction, as shown in Figure 5, the dispersion of articles analyzed on DT, DL and Explanatory Models, and most of the articles found (47 articles) refer to DT, eight articles refer to DL, and only two articles refer to Explanatory Models.

**Figure 6.**
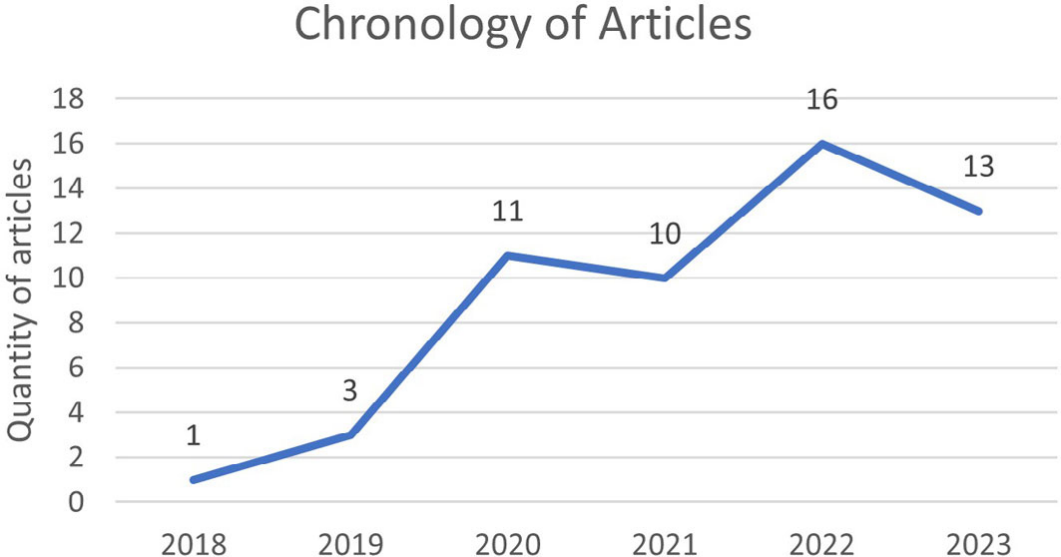
Chronology of selected articles.

**Figure 7.**
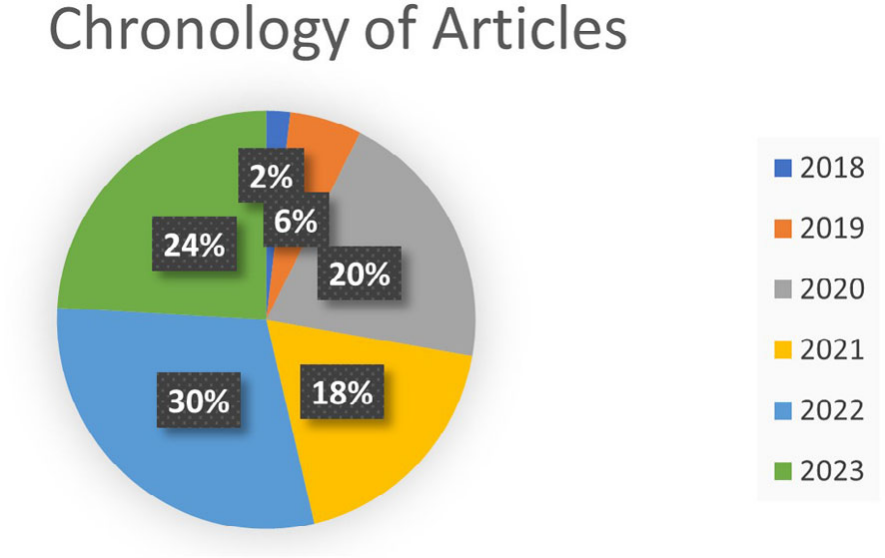
Chronology of selected articles.

According to Figure 6, the chronology of the selected articles on the research concepts is observed, as shown in Figure 7 percent of them.

After analyzing the selected articles and applying the inclusion criteria using the defined scale to validate the quality of the articles, 54 articles were obtained. Table 6 presents the authors, year, and place of publication of the analyzed articles.

**Table 6.** Articles analyzed.

	AUTHOR	YEAR	PUBLICATION
1	P. Fehér; Z. Szabó	2018	International Conference on Software, Knowledge, Information Management & Applications (SKIMA)
2	Francisco, E., Ferreira, H., Goulart, I.	2019	Texto Livre
3	S. Shin; Z. M. Rakhmatullayev	2019	International Conference on Advanced Communications Technology, ICACT
4	Dee Lemos Santos F.M., Vasconcelos A., Tribolet J., Viana P.	2019	ICEIS 2019 - Proceedings of the 21st International Conference on Enterprise Information Systems
5	Inmaculada Aznar-Díaz, Francisco-Javier Hinojo-Lucena, María-Pilar Cáceres-Reche, José-María Romero-Rodríguez	2020	Computers and Education
6	Ivanova M., Putintseva N.	2020	ACM International Conference Proceeding Series
7	Datta P., Walker L., Amarilli F.	2020	Journal of Information Technology Teaching Cases
8	Tangi L., Janssen M., Benedetti M., Noci G.	2020	Lecture Notes in Computer Science (including subseries Lecture Notes in Artificial Intelligence and Lecture Notes in Bioinformatics)
9	Alvarenga A., Matos F., Godina R., Matias J.C.O.	2020	Sustainability (Switzerland)
10	Bousdekis A., Kardaras D.	2020	Proceedings - 2020 IEEE 22nd Conference on Business Informatics, CBI 2020
11	Bower L.L.	2020	ACM International Conference Proceeding Series
12	Datta P.	2020	Communications of the Association for Information Systems
13	Gabryelczyk R.	2020	Information Systems Management
14	Jeffrey J. Pittaway, Ali Reza Montazemi	2020	Government Information Quarterly
15	Balashov A., Barabanov A., Degtereva V., Ivanov M.	2020	ACM International Conference Proceeding Series
16	I. A. Bykov; M. V. Medvedeva	2021	Proceedings of the 2021 Communication Strategies in Digital Society Seminar, ComSDS 2021
17	Schneider, S., Kokshagina, O.	2021	Creativity and Innovation Management
18	Blyznyuk A., Melnyk I., Hrinchenko Y., Solomko A., Lernyk S., Moshak O.	2021	Journal of Information Technology Management
19	Hanelt, A., Bohnsack, R., Marz, D., Antunes Marante, C.	2021	Journal of Management Studies
20	Viana A.C.A.	2021	Revista Eurolatinoamericana de Derecho Administrativo
21	Zanutto, O.	2021	International Journal of Care and Caring
22	O. Surnin; A. Stolbova; P. Sitnikov; I. Efanov; A. Ivaschenko; N. Ilyasova	2021	Proceedings of ITNT 2021-7th IEEE International Conference on Information Technology and Nanotechnology
23	Boban M., Klaric M.	2021	2021 44th International Convention on Information, Communication and Electronic Technology, MIPRO 2021 - Proceedings
24	Glavas J., Uroda I., Mandic B.	2021	2021 44th International Convention on Information, Communication and Electronic Technology, MIPRO 2021 - Proceedings
25	G. M. Jonathan; K. S. Hailemariam; B. K. Gebremeskel; S. D. Yalew	2021	2021 IEEE 12th Annual Information Technology, Electronics and Mobile Communication Conference, IEMCON 2021
26	Romero-Rodríguez, J.-M., Marín-Marín, J.-A., Hinojo-Lucena, F.-J., Gómez-García, G.	2022	Social Science Computer Review
27	Stofkova, J., Poliakova, A., Stofkova, K.R., Malega, P., Krejnus, M., Binasova, V., Daneshjo, N.	2022	Sustainability (Switzerland)
28	Rêgo, B.S., Jayantilal, S., Ferreira, J.J., Carayannis, E.G.	2022	Journal of the Knowledge Economy
29	Ahn M.J., Chen Y.-C.	2022	Government Information Quarterly
30	Park, J.-Y., Chung, D.R.	2022	Korean Journal of Internal Medicine
31	Scupola A., Mergel I.	2022	Government Information Quarterly
32	Grgurevic D., Sosko G.B., Buntak K., Kurti F.	2022	International Journal for Quality Research
33	Galushi L.T., Malatji T.L.	2022	Academic Journal of Interdisciplinary Studies
34	Alam, K., Ali, M.A., Erdiaw-Kwasie, M.O., Murray, P.A., Wiesner, R.	2022	Sustainability (Switzerland)
35	A. Pariyasiri	2022	International Conference on Digital Government Technology and Innovation, DGTi-Con 2022 - Proceedings
36	Rupeika-Apoga, R., Bule, L., Petrovska, K.	2022	Journal of Risk and Financial Management
37	Tanushev, C.	2022	Economic Alternatives
38	Lee, H., Lim, J.-A., Nam, H.-K.	2022	International Journal of Environmental Research and Public Health
39	Antunes R.	2022	ACM International Conference Proceeding Series
40	Yu, J., Wang, J., Moon, T.	2022	Sustainability (Switzerland)
41	Farias-Gaytan, S., Aguaded, I., Ramirez-Montoya, M.-S.	2022	Education and Information Technologies
42	Sergei, T., Arkady, T., Natalya, L., Pathak, R.D., Samson, D., Husain, Z., Sushil, S.	2023	Australian Journal of Management
43	Kuhlmann S., Heuberger M.	2023	Public Money and Management
44	Stoumpos, A.I., Kitsios, F., Talias, M.A.	2023	International Journal of Environmental Research and Public Health
45	Fernández, A., Gómez, B., Binjaku, K., Meçe, E.K.	2023	Education and Information Technologies
46	Frățilă A., Păunescu M., Nichita E.-M., Lazăr P.	2023	Journal of Business Economics and Management
47	Shenkoya, T., Kim, E.	2023	Sustainability (Switzerland)
48	Razak, N.A., Rasli, R.M., Ahmad, N.A., Malik, S.	2023	International Journal of Evaluation and Research in Education
49	Irani Z., Abril R.M., Weerakkody V., Omar A., Sivarajah U.	2023	Government Information Quarterly
50	Niță, V., Guțu, I.	2023	International journal of environmental research and public health
51	Al-Alawi, A.I., Messaadia, M., Mehrotra, A., Sanosi, S.K., Elias, H., Althawadi, A.H.	2023	Arab Gulf Journal of Scientific Research
52	Crișan, E.L., Marincean, A.	2023	Information Systems and e-Business Management
53	Zhou, L., Xia, Q., Sun, H., Zhang, L., Jin, X.	2023	Sustainability (Switzerland)
54	Ali, A., Raza, A.A., Qazi, I.A.	2023	Development Engineering

The number of citations of the articles analysed are those shown in Table 7.

**Table 7.** List of articles (citations).

PUBLICATION	NUMBER OF QUOTES	NUMBER OF ARTICLES
2021 44th International Convention on Information, Communication and Electronic Technology, MIPRO 2021 - Proceedings	0	2
2021 IEEE 12th Annual Information Technology, Electronics and Mobile Communication Conference, IEMCON 2021	2	1
Academic Journal of Interdisciplinary Studies	0	1
ACM International Conference Proceeding Series	1	4
Arab Gulf Journal of Scientific Research	1	1
Australian Journal of Management	0	1
Communications of the Association for Information Systems	15	1
Computers and Education	13	1
Creativity and Innovation Management	21	1
Development Engineering	0	1
Economic Alternatives	0	1
Education and Information Technologies	11	2
Government Information Quarterly	64	4
ICEIS 2019 - Proceedings of the 21st International Conference on Enterprise Information Systems	1	1
Information Systems and e-Business Management	0	1
Information Systems Management	45	1
International Conference on Advanced Communications Technology (ICACT)	12	1
International Conference on Digital Government Technology and Innovation, DGTi-Con 2022 - Proceedings	0	1
International Conference on Software, Knowledge, Information Management & Applications (SKIMA)	1	1
International Journal for Quality Research	0	1
International Journal of Care and Caring	2	1
International Journal of Environmental Research and Public Health	2	3
International Journal of Evaluation and Research in Education	0	1
Journal of Business Economics and Management	0	1
Journal of Information Technology Management	0	1
Journal of Information Technology Teaching Cases	11	1
Journal of Management Studies	261	1
Journal of Risk and Financial Management	10	1
Journal of the Knowledge Economy	9	1
Korean Journal of Internal Medicine	0	1
Lecture Notes in Computer Science (including subseries Lecture Notes in Artificial Intelligence and Lecture Notes in Bioinformatics)	16	1
Proceedings - 2020 IEEE 22nd Conference on Business Informatics, CBI 2020	11	1
Proceedings of ITNT 2021-7th IEEE International Conference on Information Technology and Nanotechnology	1	1
Proceedings of the 2021 Communication Strategies in Digital Society Seminar, ComSDS 2021	0	1
Public Money and Management	8	1
Revista Eurolatinoamericana de Derecho Administrativo	0	1
Social Science Computer Review	6	1
Sustainability (Switzerland)	51	6
Texto Livre	1	1

To provide a more detailed answer to the request questions, additional articles had to be analyzed. Due to the relevance of the topic, articles prior to 2018 were considered, as well as other articles with some relevance. These articles are described in the corresponding tables.

This study is exploratory in nature. Thus, the investigation research will have the Research Questions (RQ):

• **RQ 1**: Are there explanatory models that support DT, with a focus on DL?

Although there are still no fully established explanatory models for the relationship between DT and DL, there are theoretical approaches that support DT with a focus on DL. Some of these approaches are listed in Table 8.

• **Dynamic Capabilities Model**: This model emphasizes the importance of the culture of learning and innovation in the development of digital competencies. This suggests that organizations should adopt an iterative and experimental approach to develop digital skills, focusing on capabilities such as adaptability, innovation, and continuous learning.
• **Digital Skills Model**: This model suggests that organizations should develop a digital skills management strategy to identify, measure, and develop the skills needed for DT. This emphasizes the importance of training and developing digital skills for employees and the need to align digital skills with the organization’s strategic objectives.
• **Digital Ecosystem Model**: This model suggests that organizations should adopt a collaborative and open approach to DT, involving external partners, customers, and other stakeholders. It emphasizes the importance of the co-creation of value and collaboration in the development of digital competences and innovation.

**Table 8.** Approaches supporting DT in DL.

MODEL	AUTHOR	PUBLICATION
**DYNAMIC CAPABILITIES MODEL**	Teece (2016)	Dynamic capabilities and entrepreneurial management in large organizations: Toward a theory of the (entrepreneurial) firm
	Makadok et al. (2018)	A practical guide for making theory contributions in strategic management
	Alam et al. (2013)	Relationships between innovation capabilities, business performance, marketing performance and financial performance: A literature review
	Chesbrough & Vanhaverbeke (2018)	Open innovation and Public Policy in the EU with implications for SMEs
	Winter (2018)	Pisano on dynamic capability: why size matters
**DIGITAL SKILLS MODEL**	Vuorikari et al. (2022)	DigComp 2.2: The Digital Competence Framework for Citizens-With new examples of knowledge, skills and attitudes
	Fenech et al. (2019)	The changing role of human resource management in an era of digital transformation
	Mehrvarz et al. (2021)	The mediating role of digital informal learning in the relationship between students’ digital competence and their academic performance
	Pirola et al. (2020)	Digital readiness assessment of Italian SMEs: a case study research
	Selimović et al. (2021)	Digital workplace transformation in the financial service sector: Investigating the relationship between employees’ expectations and intentions
	Hernandez-de-Menendez et al. (2020)	Competencies for industry 4.0
**DIGITAL ECOSYSTEM MODEL**	Parker et al. (2016)	Platform revolution: How networked markets are transforming the economy and how to make them work for you
	Adner & Kapoor (2016)	Innovation ecosystems and the pace of substitution: Re-examining technology S-curves
	Schreieck et al. (2017)	The Platform Owner’s Challenge to Capture Value-Insights from a Business-to-Business IT Platform
	Van Alstyne et al. (2016)	Pipelines, platforms, and the new rules of strategy

These models can provide a useful starting point for organizations wishing to promote DL in their DT. However, it is important to remember that every organization is unique and may need to adapt these theoretical approaches to meet their specific needs.

As mentioned, there are still no established explanatory models that can explain the relationship between DT and DL, which means that research in this area is fundamental for organizations to better understand how DT affects DL and how they can promote DL in their daily tasks. The survey can help organizations develop effective strategies to improve DL among their employees and ensure that digital technologies are used in the best possible way to meet business needs. In summary, the relationship between DT and DL is crucial to the success of organizations in the digital age, and research is critical to better understand this relationship. I believe that the lack of established explanatory models can make it difficult to understand the impacts of DT on DL, as these models can help clarify how different variables and factors relate to each other and how DT can affect DL. They can also help predict the possible consequences of adopting digital technologies and identify areas in which DL needs to be improved.

• **RQ2**: What methodologies support the implemented processes involving DT?

Digital transformation has been one of the main trends in organizations in recent years. It involves the integration of digital technologies in all aspects of business, including internal processes, customer relationships, products, and services, among others. To implement this transformation successfully, it is essential to develop appropriate methodologies. Methodologies may vary depending on the context of the organization and its specific needs. In the literature, some authors specify these methodologies as understanding employee needs and developing employee-centric solutions, while others approach the methodology as iterative and incremental to develop solutions, which emphasizes collaboration and continuous delivery of value to the customer. There are also authors who advocate continuous process improvement, eliminating waste, and maximizing the value delivered to the customer. All of these approaches can be combined or adapted to meet the needs of organizations. It is important to have a clear and efficient process to manage DT and ensure its successful implementation.

Table 9 shows some methodologies that can be used to support DT processes. It is important to remember that each organization is unique and may require different approaches, depending on its specific needs. Some methodologies are commonly used to support DT processes. The methodologies mentioned above are frequently used in DT projects because they have demonstrated success in a variety of situations. Each methodology has its own advantages and specific approaches; however, they all share the ability to support DT in different ways.

• **Agile**: The agile methodology is often used in DT projects to enable an iterative and incremental approach to delivering results. This helps ensure that teams quickly adapt to changing business and market needs ( Ionel, 2009).
• **Design Thinking**: This approach focuses on the user and understanding their needs, priorities, and challenges. Teams can develop more user-centric solutions using a series of techniques and tools ( Lewrick et al., 2020).
• **Lean**: This methodology emphasizes eliminating waste and increasing efficiency, thereby making the DT process more agile and effective. It focuses on creating value for the customer and reducing the lead and cycle times ( Staats & Upton, 2009).
• **Six Sigma**: This methodology emphasizes the elimination of defects and continuous process improvement. It can be used to help organizations identify areas for improvement and implement changes to ensure more efficient and effective DT ( Tjahjono et al., 2010).

**Table 9.** Summary of DT methodologies.

AUTHOR	METHODOLOGIES			
	Agile	Design Thinking	Lean	Six Sigma
Dee et al. (2019)	X			
Feher & Szabo (2019)	X			
Francisco et al. (2019)	X			
Shin & Rakhmatullayev (2019)	X			
Balashov et al. (2020)			X	
Bousdekis & Kardaras (2020)		X		
Datta (2020)				X
Datta et al. (2020)				X
Nadkarni & Prügl (2020)	X			
Pittaway & Montazemi (2020)	X			
Tangi et al. (2020)				X
Blyznyuk et al. (2021)				X
Boban & Klaric (2021)			X	
Glavas et al. (2021)	X			
Jonathan et al. (2021)	X			
Surnin et al. (2021)			X	
Zanutto (2021)		X		
Ahn & Chen (2022)				X
Antunes (2022)		X	X	
Farias-Gaytan et al. (2022)	X			
Galushi & Malatji (2022)		X		
Grgurevic et al. (2022)	X		X	
Lee et al. (2022)	X			
Park, et al. (2022)		X		
Rêgo et al. (2022)	X	X		
Scupola & Mergel (2022)	X			
Stofkova et al. (2022)			X	
Yu et al. (2022)				X
Fernández et al. (2023)	X	X		
Kuhlmann & Heuberger (2023)	X			
Sergei et al. (2023)	X			

Studies on DT may not focus on a specific methodology but on a broader, integrated approach that combines several methodologies and strategies.

DT is a continuous and dynamic process that allows organizations to meet ever-changing demands. However, successful adoption of these technologies depends on several factors, including the accessibility of the technology, adaptability of organizations to changes arising from the adoption of the technology, and availability of specialized personnel to implement and maintain the technology. If these factors are not properly considered, the expected benefits of adopting the technology may not be guaranteed and may even harm the organization. Therefore, it is important for organizations to carefully analyze the factors involved in the adoption of new technologies before making decisions.

However, it is important to note that the size of an organization is not the only factor to consider when discussing organizational change. Other factors, such as organizational culture, financial resources, and the DL of employees can also play an important role in the success of DT. In addition, DT is not a single and continuous process, but a journey of evolution that requires constant monitoring, adjustments, and updates using the most appropriate methodology.

• **RQ3**: What are the research perspectives on explanatory models involving DT and DL as the primary factors?

There are several research perspectives in the literature on the explanatory models of DT and DL. some of these perspectives are include in Table 10.

• **Models of technology adoption**: These models examine why people do or do not adopt digital technologies and how DL can influence that process.
• **Digital empowerment models**: These focus on people’s ability to understand, evaluate, and use digital information critically and effectively.
• **Digital governance models**: These models examine how organizations manage and regulate the use of digital technologies, and how DL can affect these processes.
• **Digital innovation models**: These models focus on how organizations can use DL to drive innovation and DT.

**Table 10.** Perspectives involving DT with DL.

MODELS	AUTHOR	PUBLICATION
**MODELS OF TECHNOLOGY ADOPTION**	Venkatesh et al. (2016)	Unified theory of acceptance and use of technology: A synthesis and the road ahead
	Wejnert (2002)	Integrating models of diffusion of innovations: A conceptual framework
	Zhang et al. (2012)	A meta-analysis of mobile commerce adoption and the moderating effect of culture
	Blut & Wang (2020)	Technology readiness: a meta-analysis of conceptualizations of the construct and its impact on technology usage
**DIGITAL EMPOWERMENT MODELS**	Gilster (1997)	Digital Literacy
	Pettersson (2018)	On the issues of digital competence in educational contexts – a review of literature
	Ferrari & Punie (2013)	DIGCOMP: A framework for developing and understanding digital competence in Europe
	Zhao et al. (2021)	Digital competence in higher education research: A systematic literature review
	Bilbao et al. (2021)	A systematic literature review about the level of digital competences defined by DigCompEdu in higher education
**DIGITAL GOVERNANCE MODELS**	Spanaki & Papazafeiropoulou (2016)	The implementation of governance, risk, and compliance IS: Adoption lifecycle and enterprise value
	Shubha (2017)	Leading digital transformation with e-Governance competency framework
	Mergel et al. (2021)	Digital transformation of the German state
	Homburg (2018)	ICT, e-government and e-governance: bits & bytes for public administration
	Alvarenga et al. (2020)	Digital transformation and knowledge management in the public sector
**DIGITAL INNOVATION MODELS**	Westerman et al. (2014)	Leading digital: Turning technology into business transformation
	Vaska et al. (2021)	The digital transformation of business model innovation: A structured literature review
	Dodgson et al. (2008)	The management of technological innovation: strategy and practice
	Chawla & Goyal (2022)	Emerging trends in digital transformation: a bibliometric analysis
	Ciampi et al. (2021)	Exploring the impact of big data analytics capabilities on business model innovation: The mediating role of entrepreneurial orientation
	Feroz et al. (2021)	Digital transformation and environmental sustainability: A review and research agenda

All these research perspectives on explanatory models involve DT and DL. This field is constantly evolving, and new research perspectives are emerging as technology and society continue to change.

Challenges to overcome include resistance to change, which is one of the main obstacles facing DT. On the other hand, COVID-19 has also opened a range of opportunities for innovation and transformation in organizations, allowing them to take advantage of ICT to adapt to an increasingly digitized world. In addition, sharing digital skills between employees can contribute to the creation of a more collaborative and creative work environment in which new ideas and solutions are developed together.

In the era of DT, technology has become an integral part of business operations and the success of an organization depends on the ability of employees to use digital tools and processes. Digital transformation cannot be achieved overnight and requires cultural change within organizations as well as a change in the mindset of employees to accept and embrace this change. Therefore, education and training in digital skills are essential to help organizations and their employees face the challenges of DT and adapt to new ways of working. Digital Literacy is an essential skill for the success of DT and should be valued by organizations; however, employees may not be prepared for this change, making a careful and planned approach to DT essential.

Thus, DT involves a change in mindset towards the use of technology, and people are essential for the success of this process. It is important for organizations to provide their employees with the necessary resources and training so that they can adapt to technological changes and incorporate new tools into their work routines. In addition, cultural and organizational changes must be encouraged so that employees feel motivated to adopt new work practices. Digital transformation should not be seen only as a technological issue, but also as a holistic change that affects the culture, processes, and people within organizations.

The adoption of new technologies and methodologies in organizations can lead to the need to update the capabilities and skills of employees. Digital transformation has a significant impact on the way organizations operate, and for them to take full advantage of new technologies and methodologies, it is important that their employees are trained to work with them. As organizations digitally transform, employees must learn to use new digital tools and work with new systems and processes. This may require them to develop new capabilities, such as the ability to analyze data. Digital transformation can also create new opportunities for employees, such as working on new projects and in different areas of the organization. To ensure that employees are prepared for DT, organizations need to invest in training and education programs that help them develop the necessary skills and competencies. This should be ongoing and regularly updated to reflect changes in the ever-evolving digital environment.

Finally, through a comparative analysis of all articles researched and selected, it appears that DL is a very important factor in organizations for the success of DT, being its crucial relationship within organizations.

• **RQ4**: Which factors or variables impact the relationship between DT and DL among local public administration employees?

Studies have addressed several factors and variables that influence the relationship between DT and DL among local public administration employees. These include access to technological resources and infrastructure, availability of training and capacity building in digital environments, organizational culture and leadership support, resistance to change, collaborative work environments, and policies and regulations related to digital technology. In addition, variables such as employees’ educational level, previous experience with digital technologies, level of DL, continuous training, technology adoption, job satisfaction, role, and age were also addressed.

By addressing these factors and variables, we hope to gain a deeper understanding of the factors that influence the relationship between local public administration employees’ DL and DT. Some factors that may impact the relationship between DT and the DL of local public administration employees are included in Table 11.

• **Access to resources and technological infrastructure**: This refers to the availability of digital resources, such as computers, software, internet connectivity, and appropriate systems. Easy and reliable access to these resources facilitates employees’ DL, enabling them to effectively use digital technologies.
• **Training and capacity building in digital environments**: The existence of training and capacity-building programs in digital environments is crucial for improving employees’ DL. Appropriate training provides the knowledge, skills, and practices necessary for employees to use digital technologies effectively in their daily tasks.
• **Organizational culture and leadership support**: An organizational culture that values DT and provides support and guidance from leadership is essential. When organizational leaders demonstrate enthusiasm and support for DT, they positively influence employees’ DL, encouraging them to embrace and explore digital technologies.
• **Resistance to change**: Employees’ resistance to change can negatively impact DL. An unwillingness to learn new technologies and adapt to digital processes can make it difficult to use digital tools effectively and affect employees’ DL.
• **Collaborative work environment**: A work environment that promotes collaboration and digital knowledge sharing among employees is conducive to DL. The exchange of experience, questions, and mutual knowledge facilitates the acquisition and development of digital skills.
• **Policies and regulations related to digital technology**: Policies and regulations that address data security, privacy, and the ethical use of digital technology can affect employees’ DL. Clear guidance and appropriate guidelines provide the context for the responsible use of digital technologies.

**Table 11.** Factors that may impact the relationship between DT and DL.

FACTORS	AUTHOR	PUBLICATION
**ACCESS TO RESOURCES AND TECHNOLOGICAL INFRASTRUCTURE**	Asgarkhani, M. (2005)	Digital government and its effectiveness in public management reform: A local government perspective
	Bakon, K. A., Elias, N. F., & Abusamhadana, G. A. (2020)	Culture and digital divide influence on e-government success of developing countries: A literature review
	Papadomichelaki, X., & Mentzas, G. (2011)	Analysing e-government service quality in Greece
	Olphert, C. W., Damodaran, L., & May, A. J. (2005)	Towards digital inclusion-engaging older people in the ‘digital world’
**TRAINING AND CAPACITY BUILDING IN DIGITAL ENVIRONMENTS**	Kateryna, A. et al. (2020)	Digital literacy development trends in the professional environment
	Colbert, A., Yee, N., & George, G. (2016)	The digital workforce and the workplace of the future
	Çetin, E. (2021)	Digital storytelling in teacher education and its effect on the digital literacy od pre-service teachers
	Zulu, S. L., Saad, A. M., & Gledson, B. (2023)	Individual Characteristics as Enablers of Construction Employees’ Digital Literacy: As Exploration of Leaders’ Opinions
**ORGANIZATIONAL CULTURE AND LEADERSHIP SUPPORT**	Church, A. H., & Burke, W. W. (2017)	Four trends shaping the future of organizations and organization development
	Jonathan, G. M. et al. (2021)	Public sector digital transformation: challenges for information technology leaders
	Ashok, M. et al. (2021)	How to counter organizational inertia to enable knowledge management practices adoption in public sector organizations
	McCarthy, P., Sammon, D., & Alhassan, I. (2021)	Digital transformation leadership characteristics: A literature analysis
**RESISTANCE TO CHANGE**	Scholkmann, A. B. (2021)	Resistance to (digital) change: Individual, systemic and learning-related perspectives
	Vial, G. (2021)	Understanding digital transformation: A review and a research agenda
	Basyal, D. K., & Wan, P. D. J. (2020)	Employees’ resistance to change and technology acceptance in Nepal
	Goldman, S. R., & Scardamalia, M. (2013)	Managing understanding, applying, and creating knowledge in the information age: Next-generation challenges and opportunities
**COLLABORATIVE WORK ENVIRONMENT**	Foerster-Metz, U. S. et al. (2018)	Digital transformation and its implications or organizational behaviour
	Appio, F. P. et al. (2021)	Digital transformation and innovation management: A synthesis of existing research and an agenda for future studies
	Shin, D., & Konrad, A. M. (2017)	Causality between high-performance work systems and organizational performance
	Schneckenberg, D., Truong, Y., & Mazloomi, H. (2015)	Microfoundations of innovative capabilities: The leverage of collaborative technologies on organizational learning and knowledge management in a multinational corporation
**POLICIES AND REGULATIONS RELATED TO DIGITAL TECHNOLOGY**	Al-Sebie, M. (2014)	Organizational challenges facing integrating e-government systems: an empirical study
	Asgarkhani, M. (2005)	Digital government and its effectiveness in public management reform: A local government perspective
	Weerakkody, V. et al. (2016)	Digitally-enabled service transformation in the public sector: The lure of institutional pressure and strategic response towards change
	Misuraca, G., Barcevicius, E., & Codagnone, C. (2020)	Shaping Digital Government Transformations in the EU

Some variables that may impact the relationship between DT and the DL of local public administration employees are included in Table 12.

• **Digital literacy level**: Refers to the ability of employees to effectively use digital technologies to perform their tasks and achieve organizational goals. A high DL level indicates proficiency and confidence in using digital tools.
• **Technology adoption**: Reflects the degree to which employees adopt and incorporate digital technologies into their work routines and organizational processes. A high adoption rate indicated a positive relationship between DT and DL.
• **Job satisfaction**: This relates to employee satisfaction with the use of digital technologies in the workplace. Employees who feel satisfied with the use of digital tools may indicate a positive relationship between DT and DL.
• **Age**: Employees’ age can influence DL, with different levels of digital familiarity and skills between different age groups.
• **Previous experience**: Employees’ prior experiences with digital technologies can affect their DL. Those with previous experience may have an advantage in adopting and using digital tools.
• **Educational level**: Employees’ educational level can influence DL, and evidence suggests that people with higher educational levels tend to have better digital skills.
• **Role/Department**: The role of department employees can affect their DL, as different departments may have specific digital needs and requirements.
• **Ongoing training**: The availability of technology training opportunities can be an important factor in improving employees’ DL over time.

**Table 12.** Variables that may impact the relationship between DT and DL.

VARIABLES	AUTHOR	PUBLICATION
**DIGITAL LITERACY LEVEL**	Peng, D., & Yu, Z. (2022)	A literature review of digital literacy over two decades
	Jamil, M. I. M., & Almunawar, M. N. (2021)	Importance of Digital Literacy and Hindrance Brought About by Digital Divide
	Oh, S. S. et al. (2021)	Measurement of digital literacy among older adults: systematic review
	Tinmaz, H. et al. (2022)	A systematic review on digital literacy
**TECHNOLOGY ADOPTION**	Park, N. et al. (2009)	User acceptance of a digital library system in developing countries: An application of the Technology Acceptance Model
	Straub, E. T. (2009)	Understanding technology adoption: Theory and future directions for informal learning
	Morris, M. G., & Venkatesh, V. (2000)	Age differences in technology adoption decisions: Implications for a changing work force
	Taherdoost, H. (2018)	A review of technology acceptance and adoption models and theories
**JOB SATISFACTION**	Cetindamar, D., Abedin, B., & Shirahada, K. (2021)	The role of employees in digital transformation: a preliminary study on how employees’ digital literacy impacts use of digital technologies
	Savić, D. (2020)	COVID-19 and work from home: Digital transformation of the workforce
	Fleischer, J., & Wanckel, C. (2023)	Job satisfaction and the digital transformation of the public sector: The mediating role of job autonomy
	Nagel, L. (2020)	The influence of the COVID-19 pandemic on the digital transformation of work
**AGE**	Manor, S., & Herscovici, A. (2021)	Digital ageism: A new kind of discrimination
	Lagacé, M. et al. (2015)	How ageism contributes to the second-level digital divide: The case of Canadian seniors
	Choi, E. Y. et al. (2020)	Does perceived ageism widen the digital divide? And does it vary by gender?
	Mannheim, I. et al. (2023)	The Role of Ageism in the Acceptance and Use of Digital Technology
**PREVIOUS EXPERIENCE**	Agarwal, R., & Prasad, J. (1997)	The role of innovation characteristics and perceived voluntariness in the acceptance of information technologies
	Taherdoost, H. (2018)	A review of technology acceptance and adoption models and theories
	Tarhini, A., Arachchilage, N. A. G., & Abbasi, M. S. (2015)	A critical review of theories and models of technology adoption and acceptance in information system research
	Venkatesh, V., Thong, J. Y., & Xu, X. (2012)	Consumer acceptance and use of information technology: extending the unified theory of acceptance and use of technology
**EDUCATIONAL LEVEL**	Lee, S. H. (2014)	Digital literacy education for the development of digital literacy
	Boechler, P., Dragon, K., & Wasniewski, E. (2014)	Digital literacy concepts and definitions: Implications for educational assessment and practice
	Spires, H. A., Paul, C. M., & Kerkhoff, S. N. (2019)	Digital literacy for the 21st century
	Zhao, M., Liao, H. T., & Sun, S. P. (2020)	An education literature review on digitization, digitalization, datafication, and digital transformation
**ROLE/DEPARTMENT**	Bharadwaj, A. et al. (2013)	Digital business strategy: toward a next generation of insights
	Schwertner, K. (2017)	Digital transformation of business
	Zhang, J., & Chen, Z. (2023)	Exploring Human Resource Management Digital Transformation in the Digital Age
	Aarons, G. A., Hurlburt, M., & Horwitz, S. M. (2011)	Advancing a conceptual model of evidence-based practice implementation in public service sectors
**ONGOING TRAINING**	Kateryna, A. et al. (2020)	Digital literacy development trends in the professional environment
	Oliveira, K. K. D. S., & de SOUZA, R. A. (2022)	Digital transformation towards education 4.0
	Abd-Rabo, A., & Hashaiked, S. (2021)	The digital transformation revolution
	Manana, T., & Mawela, T. (2022)	Digital Skills of Public Sector Employees for Digital Transformation

In addition to the factors and variables described above, other control variables can also be considered, such as the demographic and organizational characteristics of employees. These variables may influence the relationship between DT and DL. These additional factors and variables can provide a more comprehensive view of the elements that may influence the relationship between DT and DL among local public administration employees.

## 4. Discussion

The relationship between DT and DL of local public administration employees was analyzed based on different factors and variables. The findings reveal important aspects that can influence employees’ DL and guide research in this field. One of the key factors is the access to technological resources and infrastructure. The availability of devices, Internet connectivity, and other adequate resources have been identified as elements that positively impact DL. On the other hand, a lack of access to these resources can hinder the development of necessary digital skills.

Training and capacity building in digital environments also play crucial roles in DL. Effective training and capacity-building programs have been associated with improvements in employee DL. However, the lack of learning and development opportunities is a significant barrier. Organizational culture and leadership support were identified as influential factors in DL. An organizational culture that values innovation, collaboration, and continuous learning encourages employee DL. Leadership support is key to promoting the adoption of digital technologies and the development of necessary skills.

Resistance to change has emerged as a challenge to overcome in improving the DL. Effective strategies to address resistance to change are needed to promote employee DL and ensure the successful adoption of digital technologies. A collaborative work environment has been highlighted as a facilitator of DL development. An environment that promotes knowledge exchange, collaboration, and cooperation among employees contributes to enhancing digital skills. Policies and regulations related to digital technology also affect employees’ DL. Clear policies and well-defined regulations are essential to provide guidance and establish guidelines for the use of digital technologies. A lack of adequate policies can create uncertainty and hinder DL development.

These findings highlight the importance of access to technological resources and infrastructure, adequate training and capacity building, favorable organizational culture, overcoming resistance to change, a collaborative work environment, and appropriate policies to promote the DL of local government employees. They provide insights into the factors that influence DL and highlight key areas that require further research.

Several research directions can be explored in studying the relationship between DT and DL of local public administration employees.

1. To assess the impact of individual variables such as age, previous experience, educational level, and function/department on the DL of local public administration employees.
  • How does employees’ age influence their willingness and ability to adopt digital technologies and develop DL?
  • Is there a correlation between employees’ previous experience with technology and their ability to adapt to DT and improve their DL?
  • What is the role of employees’ educational level in acquiring digital skills, and how does this affect DL?
  • Is there a difference in DL between different functions/departments in local public administration and how can this be addressed?
2. To investigate the relationship between employees’ DL and technology adoption, job satisfaction, and current DL levels.
  • Is there a correlation between employees’ DL level and their job satisfaction?
  • How does employee DL influence their willingness to adopt new technologies and promote DT in their daily tasks and activities?
  • To what extent does the current level of employee DL affect the effectiveness of training and capacity-building initiatives in digital environments?
3. Explore the influence of methodologies such as Agile, Design Thinking, Lean and Six Sigma in promoting DL and supporting DT in local public administration.
  • How can agile methodologies, such as agile, be applied to boost the DL of local public administration employees?
  • To what extent can the Design Thinking approach help identify employees’ DL needs and develop user-centered solutions?
  • How can Lean principles be used to optimize processes and improve employees’ DL?
  • What is the role of Six Sigma in continuously improving employee DL and reducing knowledge gaps?
4. Investigate digital governance best practices in local public administration and how they affect employee DL. This research explores the following:
  • How do digital governance policies and regulations impact employees’ DL?
  • What are the best digital governance strategies that promote effective DL?
  • How can digital governance be aligned with DT initiatives to support the development of employee DL?

These research directions address key aspects of the relationship between DT and DL among local public administration employees, allowing for further analysis and providing relevant insights for research.

Several open questions make this research on the relationship between DT and DL of local public administration employees highly relevant:

1. What is the specific impact of DT on operational efficiency and public service delivery by the local public administration? It is important to investigate how DT affects the efficiency of processes and delivery of public services.
2. What are the main challenges faced by local public administration when dealing with resistance to change during DT, and how can these challenges be overcome? Resistance to change is a common obstacle in DT. It is important to understand the specific challenges faced by local public administrations and to explore strategies to overcome resistance.
3. What are the main obstacles and gaps that local public administration employees encounter when trying to improve their DL, and how can they be addressed? Identifying the obstacles and gaps in employee DL is essential for developing effective capacity-building strategies.
4. What are the best strategies and approaches for providing effective training and capacity building in digital environments for local public administration employees? Identifying the best practices for training and capacity building is crucial for boosting employee DL.

These open questions highlight the relevance and importance of this study. By addressing these knowledge gaps, this research will contribute to a more comprehensive understanding of the relationship between DT and DL in local public administration, as well as provide practical guidance for improving operational efficiency, overcoming resistance to change, addressing barriers in DL, and developing effective training and capacity-building strategies.

## 5. Conclusions and Future Research

In conclusion, SLR is carried out mainly to identify, evaluate, and interpret the studies carried out and published in the literature. Through SLR, we were able to collect evidence on the topic to be addressed, detect, and identify opportunities for research work. Therefore, it is extremely important to carry out an SLR before starting the research, as this way, it is possible to clearly assess the research area and how the research proposal can contribute to giving more value to what has already been published.

The scientific production of DT and DL in the context of organizations is a topic of interest in the scientific community. The results of this investigation clearly show that studies predominating DT are currently a topic with some relevance. DT and its impact on DL are topics of interest to organizations, as influencing processes imply proper functioning. In this investigation, interest in these concepts over the last five years is evident. The adoption of new technologies and methodologies in organizations impacts the digital training of their employees, as it requires them to develop new digital skills to be prepared for DT.

Table 13 presents some of the main conclusions and results of the SLR for DT and DL.

**Table 13.** Conclusions and results found in SLR about DT and DL.

SLR about DT and DL	CONCLUSIONS AND RESULTS
The impact of DT on organizations	The reviews showed that DT has a significant impact on organizations, increasing efficiency, improving the customer experience and driving innovation
DT challenges	Many reviews highlighted the challenges of DT, such as cultural resistance, lack of digital training and information security
DL in Education	In the context of DL, the reviews highlighted the importance of including DL in formal and continuing education to empower people to use technology effectively
Digital skills	Some reviews have pointed to the existence of difference digital skills, where certain socio-economic groups have more limited digital access and knowledge than others
DL models and frameworks	Reviews have identified a variety of DL models and frameworks that help to better understand the concept and its dimensions
Changes in the workplace	The DT is redefining the skills needed in the workplace, with an increasing emphasis on digital knowledge and adaptability

This SLR research on the relationship between DT and DL, with the aim of creating an explanatory model, can stand out from previous reviews and add value for several reasons:

• The emphasis on creating an explanatory model distinguishes reviews from traditional reviews that summarize and synthesize existing findings. An explanatory model can help clarify the causal relationships and complex interactions between the concepts of DT and DL.
• This review can contribute to theory by developing a conceptual model that helps explain how DT affects DL and vice versa. This provides a solid theoretical framework for future research.
• By creating an explanatory model, we can identify gaps in the literature where the relationship between DT and DL has not been sufficiently explored. These gaps can guide future research in this area.
• The explanatory model has practical implications such as guidelines for organizations and adding a valuable practical component to the review.
• This review explores the relationship between DT and DL in specific contexts, allowing for a more contextualized analysis of the implications and results.
• In addition to developing an explanatory model, we expect to synthesize the existing evidence to support the relationships presented in the model. This will help to reinforce the conclusions and strengthen the validity of the model.

Technology has innovation as its main factor, having to adapt to its context and generate new learning. DT has had a systemic reach in organizations over the last five years, in which the development of employees’ digital skills contributes to the adoption of new technologies that support DT. It is important for organizations to generate strategies that guarantee systemic DT so that their services and processes evolve at a good pace. It is essential that the incorporation of technologies is accompanied by strategies that favor employees, thus promoting an increase in DL. This incorporation of technology has led organizations to rethink their processes, bearing in mind that technologies are a means of support and that their employees must be prepared for changes, especially digital changes.

No explanatory models have been found in the literature on the relationship between DT and DL, which makes research in this area even more relevant. Some authors, such as Eshet (2004), Jaeger et al. (2012), Cetindamar Kozanoglu, D., & Abedin, B. (2021), Sanders, C. K., & Scanlon, E. (2021), and Brown, N., & Brown, I. (2019) mention the relevance of this relationship. Eshet (2004) explored the key concepts of DL and the importance of survival skills in the digital age. Jaeger et al. (2012) discussed how digital inclusion and DL are fundamental to education and civic participation in the digital age. On the other hand, Cetindamar Kozanoglu D. and Abedin (2021) explore the importance of DL in the context of DT in organizations. Sanders K. and Scanlon (2021) investigated how DL affects digital inclusion, particularly among low-income populations. Finally, Brown N. and Brown (2019) examined the relationship between DT and digital skills including DL.

It is noteworthy that it was possible to answer the research questions, noting that DT is an emerging theme in organizations, especially in improving technology and adapting organizational processes, where DL is essential for improving and facilitating work. It is verified in the SLR that the concepts of DT and DL, as well as their relationship, allow a broad discussion on the subject, providing a deepening of the concepts related to each other, with the objective of strengthening their investigation.

Thus, there is a need to implement an explanatory model of the relationship between DT and DL. The methodological approach used in this study can serve as a model for other researchers investigating similar topics. The implementation of an explanatory model can be a crucial step in the current research and serves as a basis for future research. Some areas that could be explored in future research include the following.

• Comparing Models: Explanatory models can vary in terms of complexity and emphasis on different variables. Future research could compare different models to determine the most effective in explaining the relationship between DT and DL.
• Longitudinal Studies: Investigating how the relationship between DT and DL evolves over time can be an interesting area of research. Longitudinal studies can help identify the trends, changes, and factors that influence this relationship over time.
• Case Analysis: Future research could focus on detailed case studies of local public administration organizations that have undergone successful digital transformation. This would allow for a deeper analysis of the practices and strategies that have led to positive DL results.
• Effects of DL on Local Public Administration: In addition to understanding how DT affects DL, it is important to investigate how employee DL affects local public administration in terms of efficiency, quality of services, and innovation. This is an area that could be explored in future research.
• Impact on Society: In addition to the effects on public administration, investigating how the relationship between DT and DL impacts society in general could be a relevant area of research. How does improving the DL of local public administration employees affect citizen participation and delivery of public services?

There are just a few suggestions for future research that can build on the explanatory model that will be developed. Each of these areas of research can contribute to a deeper understanding of the relationship between DT and DL, and offer valuable insights for local public administration.

## Data Availability

All data underlying the results are available as part of the article and no additional source data are required. Figshare: The relationship between digital transformation and digital literacy - an explanatory model: Systematic literature review. Extended data – Table 1-Search strategy for techniques or methods or approaches to explanatory models.docx. https://doi.org/10.6084/m9.figshare.25331788 ( Arnaud *et al.*, 2023a). Figshare: The relationship between digital transformation and digital literacy - an explanatory model: Systematic literature review. Extended data – Table 2-Search strategy for review or survey on digital transformation. https://doi.org/10.6084/m9.figshare.25331785 ( Arnaud *et al.*, 2023b). Figshare: Working title: The relationship between digital transformation and digital literacy - an explanatory model: Systematic literature review. Extended data – Table 3-Search strategy for review or survey on digital literacy.docx. https://doi.org/10.6084/m9.figshare.25331782 ( Arnaud *et al.*, 2023c). Figshare: The relationship between digital transformation and digital literacy - an explanatory model: Systematic literature review. Extended data – Table 4-Search strategy for digital transformation and digital literacy.docx. https://doi.org/10.6084/m9.figshare.25331791 ( Arnaud *et al.*, 2023d). Figshare: The relationship between digital transformation and digital literacy - an explanatory model: Systematic literature review. Extended data – PRISMA_2020_flow_diagram.docx. https://doi.org/10.6084/m9.figshare.25331863.v1 ( Arnaud, 2024). Figshare: PRISMA checklist for ‘The relationship between digital transformation and digital literacy - an explanatory model: Systematic literature review’. https://doi.org/10.6084/m9.figshare.25331848 ( Arnaud *et al.*, 2023e). LICENCE CC0
