# Supplementary material for: The relationship between digital transformation and digital literacy - an explanatory model: Systematic literature review
Source: F1000Res. 2025 Jun 10;13:253. Originally published 2024 Apr 5. [Version 2] doi: 10.12688/f1000research.146991.2 (PMC12405857; doi:10.12688/f1000research.146991.2)
Supplement: Peer review history

# Reviewer report: Reviewer response for version 2

**DOI:** 10.5256/f1000research.183406.r403692

**Published:** 2025-09-02

**Authors:** Thomais Gkrimpizi

**Affiliations:** 1 International Hellenic University, Thermi, Greece

Dear authors,

 I really liked the draft and found the article particularly interesting. I suggest some corrections before resubmission, which I list below, and I sincerely hope that they will help you further strengthen the manuscript. I wish you every success in the editing and publication process.

 In the following lines, I share my comments on your manuscript:

 Methodology

 According to the methodology used in this article and in Figure 1, the first step is to define the research questions (RQs). The RQs form the basis for the next step and are part of the methodology. After that, it is necessary to define the search process. The next step requires the definition of inclusion and exclusion criteria. Finally, it is necessary to define the quality assessment.

 Therefore, in this section, you must include all these steps. For this reason, it is necessary to restructure the article and transfer many of the findings from the results section to the methodology section.

 Results

 In this section, you need to refer only to the results from the findings, which should be done in accordance with the research questions mentioned in the methodology (section 2).

 Discussion

 In a discussion, you should develop your own ideas, taking into account everything mentioned in the results. The abstract states: *“Based on these findings, an explanatory model was developed that maps the interaction between these variables and their impact on digital transformation outcomes”.*

 The explanatory model should be clear, which, in my opinion, is unclear.

Are the rationale for, and objectives of, the Systematic Review clearly stated?

Yes

Is the statistical analysis and its interpretation appropriate?

Yes

If this is a Living Systematic Review, is the ‘living’ method appropriate and is the search schedule clearly defined and justified? (‘Living Systematic Review’ or a variation of this term should be included in the title.)

Yes

Are sufficient details of the methods and analysis provided to allow replication by others?

Partly

Are the conclusions drawn adequately supported by the results presented in the review?

Partly

Reviewer Expertise:

digital transformation, project management. digital education

I confirm that I have read this submission and believe that I have an appropriate level of expertise to confirm that it is of an acceptable scientific standard, however I have significant reservations, as outlined above.

# Reviewer report: Reviewer response for version 1

**DOI:** 10.5256/f1000research.161124.r382360

**Published:** 2025-06-03

**Authors:** Pedro R. Palos-Sanchez

**Affiliations:** 1 Department of Financial Economy and Operation Management, Faculty of Economics and Business Sciences, University of Seville, Seville, Spain

Thank you very much for the opportunity to read your manuscript entitled: “The relationship between digital transformation and digital literacy - an explanatory model: Systematic literature review" submitted for the F1000 Journal. This paper seeks to understand how digital literacy can impact Digital Transformation in organizations and, through the construction of an explanatory model, allows the identification of variables that influence this relationship by developing strategies to improve the digital literacy of employees in organizations.

 The following are my comments, all intended to improve the final result of this research work.

**The focus of the paper**

 Overall, this is an interesting research topic, closely related to the digital transformation and digital literacy.

**Abstract**

 The abstract presents the main research objectives and methodology; however, the methodology are not shown completely (lack of number of analyzed documents); the research findings and implications are not clear.

**Introduction**

 - The explanation of originality (research gap) uses too little evidence from previous studies, and when used, these studies are too old. This leads to the concern about the research novelty, especially in recent times. For example, must cited recent works or reviews about digital transformation:

 Brazo, P., Velicia-Martín, F., Palos-Sanchez, P. R., & Rodrigues, R. G. (2023). The Effect of Coercive Digitization on Organizational Performance: How Information Resource Management Consulting Can Play a Supporting Role. *Journal of Global Information Management (JGIM)*, 31(2), 1-23. http://doi.org/10.4018/JGIM.326282

 Marino-Romero JA, Palos-Sanchez PR and Velicia-Martin FA (2023). Evolution of digital transformation in SMEs management through a bibliometric analysis. Technological Forecasting & Social Change, Volume 199, 123014 http://doi.org/10.1016/j.techfore.2023.123014 

 Brazo, Pedro; Velicia-Martín, Félix; Palos-Sánchez, Pedro; Roldán, José L. (2024). Thrive or Dive: Navigating the Waves of Mandatory Digital Transformation. *European Business Review*. 10.1108/EBR-03-2024-0097

**Theoretical part**

 - The authors should add findings in terms of topics, methods,... related to digital transformation and digital literacy that the authors has summarized from the systematic literature review.

**The aim, Methodology, and Data**

 - Research aims and research questions were presented in the Results section but specific statements on the research novelties are not well-explained. I propose to show in final introduction section and developpe in results.

**Results**

**Discussion**

 There is no link between the result part with the discussion part. Therefore, it seems that the results obtained from the study provide interesting discussions or implications. However, authors do not compare with another author or results. Probably, they need move paragraphs from conclusions (with references) to discussion.

**Conclusion**

 - The conclusion could be divided in practical and theoretical implications.

 - The authors should add the limitation of the research/results.

Are the rationale for, and objectives of, the Systematic Review clearly stated?

Yes

Is the statistical analysis and its interpretation appropriate?

Not applicable

If this is a Living Systematic Review, is the ‘living’ method appropriate and is the search schedule clearly defined and justified? (‘Living Systematic Review’ or a variation of this term should be included in the title.)

Yes

Are sufficient details of the methods and analysis provided to allow replication by others?

Yes

Are the conclusions drawn adequately supported by the results presented in the review?

Yes

Reviewer Expertise:

digital economy

I confirm that I have read this submission and believe that I have an appropriate level of expertise to confirm that it is of an acceptable scientific standard, however I have significant reservations, as outlined above.

## References

[1] : The Effect of Coercive Digitization on Organizational Performance. *Journal of Global Information Management* .2023;31(2) : 10.4018/JGIM.326282 1-23 10.4018/JGIM.326282

[2] : Evolution of digital transformation in SMEs management through a bibliometric analysis. *Technological Forecasting and Social Change* .2024;199: 10.1016/j.techfore.2023.123014 10.1016/j.techfore.2023.123014

[3] : Thrive or dive: navigating the waves of mandatory digital transformation. *European Business Review* .2025;37(3) : 10.1108/EBR-03-2024-0097 486-516 10.1108/EBR-03-2024-0097

# Reviewer report: Reviewer response for version 1

**DOI:** 10.5256/f1000research.161124.r297239

**Published:** 2024-07-18

**Authors:** Silvia Farias-Gaytan

**Affiliations:** 1 Tecnologico de Monterrey, Monterrey, Mexico

Each section is well-organized and describes each phase and results in accordance with the objective and research questions.

 An aspect that can be included as part of the conclusion is about the limitations encountered during the study, in order to serve as a reference for other researchers.

 A suggestion, that the abstract be structured following IMRDC and that the methodology used be stated because it has not been included.

Are the rationale for, and objectives of, the Systematic Review clearly stated?

Yes

Is the statistical analysis and its interpretation appropriate?

Yes

If this is a Living Systematic Review, is the ‘living’ method appropriate and is the search schedule clearly defined and justified? (‘Living Systematic Review’ or a variation of this term should be included in the title.)

Not applicable

Are sufficient details of the methods and analysis provided to allow replication by others?

Yes

Are the conclusions drawn adequately supported by the results presented in the review?

Yes

Reviewer Expertise:

educational innovation, higher education, digital transformation

I confirm that I have read this submission and believe that I have an appropriate level of expertise to confirm that it is of an acceptable scientific standard.
